# Comprehensive bioinformatics and in vitro studies reveal the carcinogenic role and molecular basis of endocrine disruptors in prostate cancer

**DOI:** 10.3389/fcell.2025.1712195

**Published:** 2025-12-03

**Authors:** Feng Yu, Bangwei Che, Wei Li

**Affiliations:** 1 Department of Urology, First Affiliated Hospital of Guizhou University of Traditional Chinese Medicine, Guiyang, China; 2 Department of Urology, The Affiliated Hospital of Guizhou Medical University, Guiyang, China

**Keywords:** endocrine disruptors, prostate cancer, network toxicology, natural active products, PLK1

## Abstract

**Background:**

In recent years, growing attention has been paid to the carcinogenicity of endocrine disruptors (EDs). However, their relationship with prostate cancer (PCa) remains unclear. This study investigates the association between EDs and PCa to identify key genes that may bridge this relationship.

**Methods:**

The ADME properties and carcinogenicity of the selected endocrine-disrupting chemicals EDs were predicted using the ADMETlab 3.0 and ProTox 3.0 platforms, respectively. Potential target genes related to EDs and PCa were obtained by integrating multiple public databases. A protein-protein interaction (PPI) network of the overlapping genes was constructed and visualized, followed by GO and KEGG enrichment analyses to explore their potential biological mechanisms. From 101 machine learning algorithm combinations, the most relevant key genes for PCa progression were screened. Molecular docking analysis was used to evaluate the binding properties between ED compounds and key targets. Pan-cancer analysis was employed to examine the general role of key genes across multiple cancer types. The Comparative Toxicogenomics Database (CTD) was used to identify natural active products potentially targeting the core genes. Finally, *in vitro* cell experiments were conducted to validate the effects of EDs on PCa cells and the intervention effects of related natural products.

**Results:**

Initially, predictions from the ADMETlab 3.0 and ProTox 3.0 platforms indicated significant *in vivo* accumulation, endocrine-disrupting effects, and carcinogenicity for the 12 common EDs. Subsequently, the integration of multiple databases identified 233 overlapping targets associated with PCa. GO and KEGG enrichment analyses revealed that these targets are primarily involved in regulating cell proliferation, inflammatory responses, and cancer cell metabolism. Among the evaluated machine learning algorithms, the CoxBoost + SuperPC hybrid model demonstrated superior predictive performance and robustness. Subsequent analysis pinpointed three key regulatory genes: CD38, MMP11, and PLK1. Molecular docking simulations confirmed potential interactions between EDs compounds and the core target, PLK1. Furthermore, five natural active products were identified as potential agents to mitigate the adverse effects induced by EDs exposure. Finally, *in vitro* cell experiments demonstrated that Benzo[a]pyrene promotes PLK1 expression and PCa progression, whereas Cryptotanshinone effectively counteracts these effects.

**Conclusion:**

This multidisciplinary study unveils PLK1 as a pivotal molecular target through which EDs drive PCa progression. Furthermore, we identify five natural compounds, notably Cryptotanshinone, that counteract the carcinogenic effects of EDs by targeting PLK1. These findings provide crucial molecular insights into ED-induced carcinogenesis and reveal promising targets for the prevention and intervention of PCa.

## Introduction

Endocrine disruptors (EDs) are exogenous chemicals or mixtures that interfere with the function of the endocrine system, causing adverse health effects in individuals, offspring, or populations (Kahn et al., 2020; Yilmaz et al., 2020; Symeonides et al., 2024). These substances typically have very low effect thresholds and are widely found in daily-use products, including plastic bottles, food can linings, epoxy-encapsulated electronics, recycled paper materials, and various food packages (Yilmaz et al., 2020; Symeonides et al., 2024). Human exposure to EDs occurs mainly through ingestion of contaminated water and food, but also through direct skin contact or inhalation of polluted air (Yilmaz et al., 2020; Symeonides et al., 2024). Most EDs, due to their structural similarity to endogenous hormones, can mimic estrogen or antagonize androgen action, interacting with estrogen receptors (ER), androgen receptors (AR), and other non-nuclear receptors, thereby disrupting multiple hormonal pathways (Kahn et al., 2020; Yilmaz et al., 2020; Symeonides et al., 2024; Macedo et al., 2023; Singh, 2024). Notably, growing research attention is being paid to the potential carcinogenic effects of EDs, particularly their role in promoting hormone-related cancers such as prostate cancer (PCa), sparking widespread concern (Macedo et al., 2023; Singh, 2024; Pang et al., 2024; Feijó et al., 2025).

PCa is a clinically heterogeneous disease that can exhibit aggressive behavior progressing to metastasis or remain asymptomatic with an indolent course. It is the most common malignancy and the second leading cause of cancer-related deaths among men worldwide, posing a major health threat and a substantial healthcare burden (Siegel et al., 2024; Teo et al., 2019). An estimated 1.4 million new cases and 396,000 deaths occurred globally in 2024 (Teo et al., 2019). However, the mechanisms behind its high incidence remain unclear. Established risk factors for PCa include age, ethnicity, and family history, yet the roles of environmental determinants, lifestyle factors, and dietary components in PCa pathogenesis are still not fully understood (Siegel et al., 2024; Yin et al., 2025). Previous studies have highlighted the crucial involvement of EDs in the development of PCa (Feijó et al., 2025; Hong et al., 2025; Zhou et al., 2025; Lacouture et al., 2022). However, current laboratory evidence remains insufficient, as earlier research has predominantly focused on the effects of individual EDs rather than comprehensively elucidating the key mechanisms within EDs-PCa interaction networks. Thus, the precise relationship between EDs and PCa has yet to be conclusively determined.

The rapid development of bioinformatics has provided important support for deciphering the complex mechanisms between environmental exposure and disease occurrence (Li Y. et al., 2025; Goldenberg et al., 2019). Utilizing multi-omics integration strategies and network analysis techniques, researchers can systematically uncover key targets and signaling pathways associated with diseases. Additionally, machine learning has shown significant advantages in disease risk prediction and core target screening. Based on this, this study integrates multimodal bioinformatics analysis, machine learning algorithms, and *in vitro* cell experiments to comprehensively investigate the potential association between EDs and PCa and to deeply explore the underlying molecular mechanisms.

## Materials and methods

### Data sources

Consistent with a previously reported study (Li W. et al., 2025), this research integrated transcriptomic data and matched clinical information from 1,028 PCa patients. The data were obtained from the following two sources: 1. the TCGA-PRAD cohort (accessed via the UCSC Xena platform), and 2. three independent cohorts from the GEO database (GSE21032, GSE70770, and GSE116918). To enhance statistical power, GSE21032 and GSE70770 were merged into a combined cohort, and technical batch effects were corrected using the ComBat algorithm from the sva package in R. To ensure data reliability, patients lacking biochemical recurrence (BCR) status or with less than 1 month of follow-up were excluded. Detailed clinical characteristics of each cohort are summarized in Supplementary Tables S1, S2.

### Toxicity prediction

Based on their environmental prevalence, significant potential for human exposure, and previous literature reports (Song et al., 2025), we selected 12 common EDs. Their toxicity profiles and relevant parameters were systematically predicted using canonical SMILES structures from the PubChem database, executed on the ProTox 3.0 (Fu et al., 2024) and ADMETlab 3.0 (Banerjee et al., 2024) platforms. These tools cover multiple toxicological endpoints, enabling a comprehensive assessment of the Absorption, Distribution, Metabolism, Excretion, and Toxicity (ADMET) properties, thereby providing a critical foundation for subsequent experimental validation and risk management.

### Identification of EDs target genes

To comprehensively identify potential targets of the 12 EDs, three authoritative databases were queried: SwissTargetPrediction, TargetNet, and PharmMapper. The canonical SMILES strings and SDF structure files of all selected EDs were obtained from the PubChem database. Target prediction was performed using SwissTargetPrediction and TargetNet, followed by target identification via the PharmMapper platform. The following thresholds were applied to screen for high-confidence human targets: probability > 0.01 for SwissTargetPrediction, AUC ≥ 0.7 for TargetNet, and conformations = 300 with an energy cutoff = 20.0 kcal/mol for PharmMapper. Predicted targets from each database were integrated to construct individual target profiles for each ED, and targets from all 12 EDs were aggregated into a unified set for subsequent analysis. During data standardization, the UniProtKB database was used to uniformly correct gene symbols and functional annotations of the predicted targets, with Entrez Gene ID used as the unique identifier.

### Acquisition of PCa targets

Transcriptomic data from 534 patients in the TCGA-PRAD cohort were analyzed, including 483 tumor tissues and 51 normal control tissues (Supplementary Table S2). Differential expression analysis was performed using the DESeq2 R package, with screening thresholds set at |log2FoldChange| > 0.65 and adjusted p-value < 0.05. Significantly differentially expressed genes were identified as potential therapeutic targets for PCa and used for further investigation.

### Identification of EDs-PCa targets and PPI network construction

Common targets between EDs and PCa were identified using Venn analysis. Subsequently, protein-protein interaction (PPI) analysis was performed on these overlapping targets using the STRING database (confidence score threshold ≥ 0.4), which integrates known and predicted protein associations, including physical interactions and functional links. After obtaining PPI data, non-essential targets were filtered out to construct a high-quality target-protein interaction network. This network was imported in TSV format into Cytoscape 3.9.0 for visualization and topological analysis. Using the built-in “Centiscape 2.0” tool in Cytoscape, multiple topological parameters—including degree centrality, closeness centrality, and betweenness centrality—were calculated to assess node importance within the network. Finally, genes were ranked based on closeness centrality, with higher values indicating greater hub importance in the network.

### Functional enrichment analysis

Gene Ontology (GO) systematically categorizes gene functions into three classes: Cellular Component (CC), Molecular Function (MF), and Biological Process (BP). The Kyoto Encyclopedia of Genes and Genomes (KEGG) links genomic information with functional pathways at a systems level. In this study, the R package clusterProfiler was used to perform GO and KEGG functional enrichment analyses on the screened targets.

### Development and evaluation of an EDs-PCa risk model using machine learning

Referring to previously established modeling workflows, this study constructed 101 combinations based on 10 machine learning algorithms to develop a high-precision diagnostic model. The TCGA-PCa cohort was used as the training set, and the GSE and GSE116918 datasets served as external validation sets. Models were ranked by average C-index, and the best-performing combination was selected as the final diagnostic model. Patients in the TCGA-PRAD, GSE, and GSE116918 cohorts were then divided into high-risk and low-risk groups based on median risk scores. Kaplan-Meier (K-M) curves were used to analyze survival differences between groups, and the predictive performance of the model was validated across the three independent datasets. The classification accuracy of the model was assessed by calculating the area under the receiver operating characteristic curve (AUC) (Xu et al., 2024).

### Molecular docking

To elucidate the interaction mechanisms between EDs and EDs-PCa-related target proteins, molecular docking simulations were performed. The molecular structures of EDs were obtained from the PubChem database, and the three-dimensional structures of target proteins were sourced from the AlphaFold protein structure database. AutoDockTools 1.5.7 was used to process molecular and protein structures and perform docking simulations to predict binding modes, binding affinity (expressed as binding free energy ΔG), and potential functional impacts. A binding free energy lower than 0 kcal/mol indicates spontaneous binding, while a value below −5.0 kcal/mol suggests a stable complex structure with strong binding capacity.

### Screening of natural active products

To identify natural active ingredients that may counteract the adverse effects of endocrine disruptors, this study systematically screened natural products related to the core target PLK1 in the EDs-PCa interaction network using the Comparative Toxicogenomics Database (CTD). The binding potential of these natural products was further validated through molecular docking.

### Cell culture and intervention

Prostate cancer cells (DU145) were purchased from the Cell Bank of the Chinese Academy of Sciences. Cells were cultured in a constant temperature incubator at 37 °C with 5% CO_2_, following the supplier’s recommended protocol. Benzo[a]pyrene and cryptotanshinone were administered at concentrations of 10 μM and 0.5 μM, respectively, following previously established protocols. (Gao et al., 2020; Xu et al., 2012).

### Western blot

Total protein was extracted from DU145 cells using RIPA lysis buffer (Solarbio, China) supplemented with a protease inhibitor cocktail (Yeasen, China). Protein samples were separated by 10% SDS-polyacrylamide gel electrophoresis and then transferred to PVDF membranes using a wet transfer method. The membranes were blocked with 5% skim milk at room temperature for 1 h, followed by incubation with an anti-PLK1 primary antibody (1:1,000 dilution; catalog #10305-1-AP, Proteintech) at 4 °C overnight. After washing with TBST, the membranes were incubated with species-appropriate secondary antibodies at room temperature for 2 h.

### EdU proliferation assay

To evaluate the effect of EDs on DU145 cell proliferation, an EdU (5-ethynyl-2′-deoxyuridine) cell proliferation detection kit (Beyotime Biotechnology, Shanghai) was used. Cells were seeded in 24-well plates at a density of 3 × 10^3^ cells per well. After 24 h of culture, 20 µL of EdU working solution was added, and the cells were incubated at 37 °C for 2 h. Cell fixation and staining were then performed according to the manufacturer’s instructions. Finally, EdU-positive cells were observed under a fluorescence microscope, and representative images were captured at ×20 magnification.

### Wound healing assay

To assess the effect of EDs on cell migration ability, treated and untreated DU145 cells were seeded in 6-well plates and cultured until full confluence was achieved. A sterile 200 µL pipette tip was used to create uniform scratches in the cell monolayer. After washing with PBS to remove detached cells, a standardized *in vitro* wound model was established. To minimize the impact of cell proliferation on experimental results, the cells were subsequently switched to a low-serum migration medium. Wound closure was observed and recorded at 0 h and 48 h post-scratching using a phase-contrast microscope (×10 objective).

### Transwell invasion assay

Cells were resuspended in serum-free medium and seeded into the upper chamber of a Transwell® insert (polycarbonate membrane, 8 µm pore size). The lower chamber was filled with RPMI-1640 medium containing 10% FBS as a chemoattractant. After 24 h of incubation at 37 °C with 5% CO_2_, cells that had migrated to the lower chamber were fixed with 4% paraformaldehyde and stained with 0.1% crystal violet. Five random fields per sample were photographed using an inverted microscope (×10 objective), and the number of migrated cells was counted.

### Statistical analysis

All statistical analyses were performed using R software (version 4.2.2). Differences in gene expression were assessed using the non-parametric Wilcoxon signed-rank test and the parametric paired Student’s t-test, with a two-sided p-value < 0.05 considered statistically significant. Disease-free survival (DFS) was analyzed using Cox proportional-hazards regression models and Kaplan-Meier curves. All *in vitro* cell experiments were independently repeated at least three times, and the data from these biological replicates were used for statistical analysis.

## Results

### Preliminary assessment of the ED toxicity network

Computational profiling using the ADMETlab 3.0 database revealed that all 12 selected EDs exhibit significant carcinogenic potential and a high propensity for bioaccumulation. This is evidenced by strong oral absorption (Caco-2 permeability Papp > 1.2 × 10^−6^ cm/s), high lipophilicity (LogP > 5), and low plasma clearance (CLplasma < 10 mL/min/kg). Further toxicological risk assessment via the ProTox 3.0 database indicated that most of these EDs possess substantial endocrine-disrupting activity and carcinogenic potential. Specifically, the majority showed a high probability of carcinogenicity (0.58–0.94) and displayed agonist or antagonist activity against various endocrine receptors, including the aryl hydrocarbon receptor, estrogen and androgen receptors, aromatase, and thyroid hormone receptors. These predictions collectively indicate a moderate to high health risk. Despite being marketed as environmentally friendly plasticizer alternatives, our findings suggest that these EDs may pose a significant threat to human health. Consequently, this study underscores the need for stringent risk assessment and control measures during their production and application. Further experimental validation is warranted to confirm these computational predictions and to explore strategies for mitigating their toxicity.

### Acquisition of EDs-PCa targets

**FIGURE 1:**
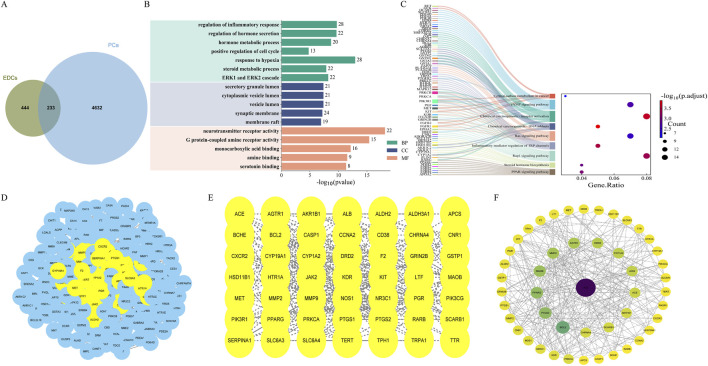
Enrichment analysis of EDs-PCa and PPI network. **(A)** Overlapping gene acquisition; **(B)** GO enrichment analysis; **(C)** KEGG enrichment analysis; **(D–F)** The construction process of PPI interaction network.

After integrating target prediction results from the aforementioned multiple databases, a total of 677 unique ED-related target genes for the 12 major EDs were identified (Supplementary Table S3). Differential expression analysis further identified 3,602 differentially expressed genes in tumor tissues (see Supplementary Table S4). Venn analysis identified 233 overlapping genes common to both EDs and PCa (Figure 1A). These genes may serve as potential therapeutic targets in ED-mediated prostate carcinogenesis.

### Enrichment analysis of EDs-PCa targets

GO functional enrichment analysis of the 233 EDs-PCa-related genes revealed significant enrichment in biological processes such as inflammatory response, cell proliferation, hypoxia response, hormone secretion, and metabolic regulation (Figure 1B). KEGG pathway analysis further indicated involvement in the PPAR signaling pathway, steroid hormone biosynthesis, Rap1 signaling pathway, TRP channels in inflammatory mediator regulation, Ras signaling pathway, chemical carcinogenesis (including DNA adduct formation and receptor activation), central carbon metabolism in cancer, and the cAMP signaling pathway, among other key pathways (Figure 1C). In summary, EDs may promote the initiation and progression of prostate cancer by interfering with inflammatory responses, cell death processes, endocrine functions, and multiple carcinogenic pathways.

### Construction of the EDs-PCa target PPI network

The 233 overlapping EDs-PCa target genes were imported into the STRING database for PPI analysis with a confidence threshold set at ≥ 0.4. After removing isolated nodes, the final network contained 226 target proteins. The PPI network was visualized using Cytoscape 3.10.3, with nodes arranged by degree; darker colors and larger diameters indicate stronger interactions within the network. Topological analysis identified five core targets within the EDs-PCa interaction network: ALB, BCL2, MAOB, PTGS2, and PPARG (Figures 1D–F). This network visualization not only clearly illustrates the interactions between key targets but also provides important clues for further exploration of the potential molecular mechanisms linking EDs and prostate cancer.

### Establishment and evaluation of an EDs-PCa prediction model

**FIGURE 2:**
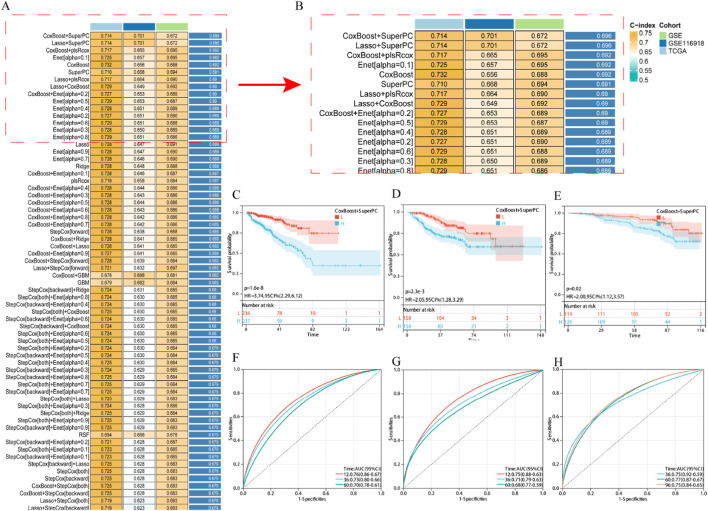
Establishment and validation of EDs PCa target related risk prediction model. **(A,B)** The average C-index of 101 robot algorithm combinations; **(C–E)** KM survival curve, **(C)** TCGA-PRAD, **(D)** GSE, **(E)** GSE116918; **(F–H)** ROC curve, **(F)** TCGA-PRAD, **(G)** GSE, **(H)** GSE116918. Note: H: Expressing an array higher than the median; L: Expressing an array lower than the median.

Univariate Cox regression analysis preliminarily identified 23 EDs-PCa genes significantly associated with DFS in the TCGA-PRAD and GSE cohorts (Supplementary Figure S1). After systematic evaluation of 101 algorithm combinations, the CoxBoost+SuperPC hybrid model was identified as having the optimal predictive performance. This model incorporated nine key genes (Figures 2A,B; see Supplementary Table S5) and achieved an average C-index of 0.696. Kaplan-Meier analysis showed that patients with high-risk scores had significantly shorter DFS in the TCGA-PRAD (Figure 2C), GSE (Figure 2D), and GSE116918 (Figure 2E) cohorts. Time-dependent receiver operating characteristic (ROC) curves further validated the model’s predictive efficacy for disease progression at different time points: in the TCGA-PRAD cohort, the 1-, 3-, and 5-year area under the curve (AUC) values were 0.77, 0.73, and 0.68, respectively (Figure 2F); in the GSE cohort, they were 0.75, 0.71, and 0.68 (Figure 2G); and in the GSE116918 cohort, the 3-, 5-, and 8-year AUC values were 0.75, 0.77, and 0.75, respectively (Figure 2H). These results highlight the significant value of this risk model, constructed from nine EDs-PCa genes, for prognostic assessment in prostate cancer.

### Survival analysis and expression validation of core targets in the EDs-PCa prediction model

**FIGURE 3:**
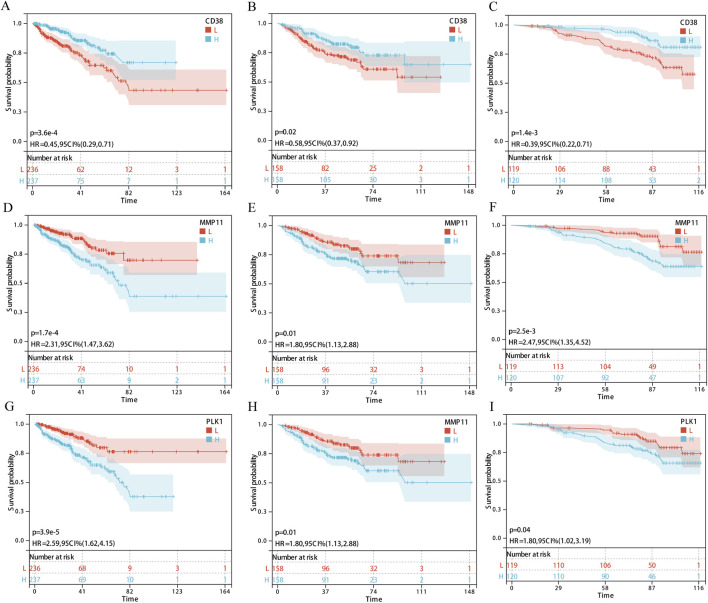
Survival analysis of core genes (CD38, MMP11, and PLK1) in three cohorts. **(A–C)** CD38; **(D–F)** MMP11; **(G–I)** PLK1; **(A,D,G)** TCGA-PRAD; **(B,E,H)** GSE; **(C,F,I)** GSE116918. Note: H: Expressing an array higher than the median; L: Expressing an array lower than the median.

**FIGURE 4:**
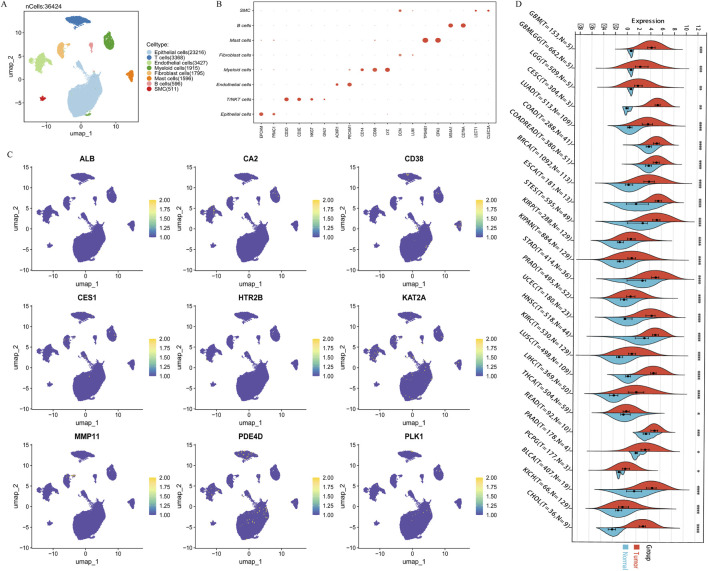
The expression and localization of core genes in the tumor microenvironment. **(A)** 7 single-cell subpopulations; **(B)** Annotation markers for 7 single-cell subpopulations; **(C)** The expression of 9 core genes in the microenvironment; **(D)** Differential expression of core gene PLK1 in 26 TCGA database tumors. Note: *p < 0.05; **p < 0.01; ***p < 0.001; ****p < 0.0001.

Kaplan-Meier analysis showed that across all three cohorts, only the expression of three out of the nine key genes, CD38 (Figures 3A–C), MMP11 (Figures 3D–F), and PLK1 (Figures 3G–I), was significantly associated with PCa DFS. CD38 expression was associated with longer DFS, while MMP11 and PLK1 were associated with shorter DFS. Immunohistochemical results from the Human Protein Atlas (HPA) database suggested decreased protein expression levels of CD38 in prostate cancer tissues (Supplementary Figures S3A,B), while MMP11 (Supplementary Figures S3C,D) and PLK1 (Supplementary Figures S3E,F) showed moderate expression in both normal and cancerous tissues. Transcriptomic analysis of 362,424 quality-controlled single cells identified seven major cell subpopulations within the tumor microenvironment (Figures 4A,B), including epithelial cells, T cells, B cells, macrophages, endothelial cells, mast cells, and smooth muscle cells. Gene expression visualization (Figure 4C) revealed distinct distribution patterns for the three key prognostic genes: MMP11 was specifically expressed in fibroblasts, while CD38 and PLK1 were widely expressed across multiple cell types. Given the established critical role of PLK1 in the development and progression of prostate cancer and its widespread expression in multiple cell populations within the microenvironment, PLK1 was selected as the core target for subsequent mechanistic exploration. Pan-cancer analysis (based on the SangerBox database) showed that PLK1 was significantly upregulated in the vast majority of cancer types (Figure 4D), and its high expression was significantly associated with poor progression-free survival in various tumors, including GBMLGG, KIPAN, KIRP, KIRC, PRAD, LGG, ACC, KICH, LIHC, PAAD, MESO, UAM, LUAD, BRCA, THCA, SKCM-M, SARC, SKCM, and BLCA (Supplementary Figure S4). These results collectively underscore the important role of PLK1 in tumorigenesis and development, suggesting it may be a key molecular target through which endocrine disruptors drive carcinogenic processes.

### Effects of the 12 EDs on the core target PLK1

Molecular docking was used to evaluate the binding interactions between the 12 EDs and the PLK1 protein. The results (Table 1) showed that all tested ED compounds could spontaneously bind to PLK1 (ΔG < 0 kcal/mol). Five compounds exhibited high binding stability (ΔG < −5 kcal/mol): Anthracene (−6.15 kcal/mol), Benzo[a]pyrene (−6.89 kcal/mol), Clofenotane (−6.27 kcal/mol), Polychlorinated biphenyls (−5.74 kcal/mol), and Triclosan (−5.09 kcal/mol). These results suggest that EDs may regulate prostate cancer-related biological processes by directly binding to PLK1. As Benzo[a]pyrene showed the highest binding affinity, it was selected for subsequent functional experimental validation.

**TABLE 1:** Binding of 12 endocrine disruptors to PLK1.

Name	Binding energy(kcal/mol)
Anthracene	−6.15
Benzo[a]pyrene	−6.89
Glyphosate	−4.68
DEHP	−2.04
DBP	−2.63
Bisphenol A	−2.04
Polychlorinated biphenyls	−5.74
Clofenotane	−6.27
PFOA	−2.13
Malathion	−2.38
Diazinon	−3.44
Triclosan	−5.09

### Screening of potentially intervening natural active products

In recent years, growing experimental evidence has shown that natural active products, due to their multi-target and multi-pathway characteristics, hold significant potential in PCa drug development. To explore potential compounds that could mitigate the adverse effects of ED exposure, this study searched the Comparative Toxicogenomics Database (CTD) for natural active components related to PLK1. Five natural products associated with PLK1 were identified: quercetin, resveratrol, curcumin, cryptotanshinone, and luteolin. Further molecular docking analysis (Table 2) indicated that all five natural active ingredients could bind stably to PLK1. Except for curcumin (−4.15 kcal/mol), the binding free energy (ΔG) of the remaining four active products was less than −5 kcal/mol, with cryptotanshinone having the lowest ΔG (−6.49 kcal/mol), suggesting a stable binding process and high affinity. These results provide an important basis and research direction for developing natural product intervention strategies to antagonize ED-related carcinogenic effects.

**TABLE 2:** Binding of 5 natural active products to PLK1.

Name	Binding energy(kcal/mol)
Caffeine	−5.05
Cryptotanshinone	−6.46
Curcumin	−4.16
Quercetin	−5.39
Resveratrol	−5.09

### Cryptotanshinone intervention ameliorates benzo[a]pyrene-induced effects on PCa cells

**FIGURE 5:**
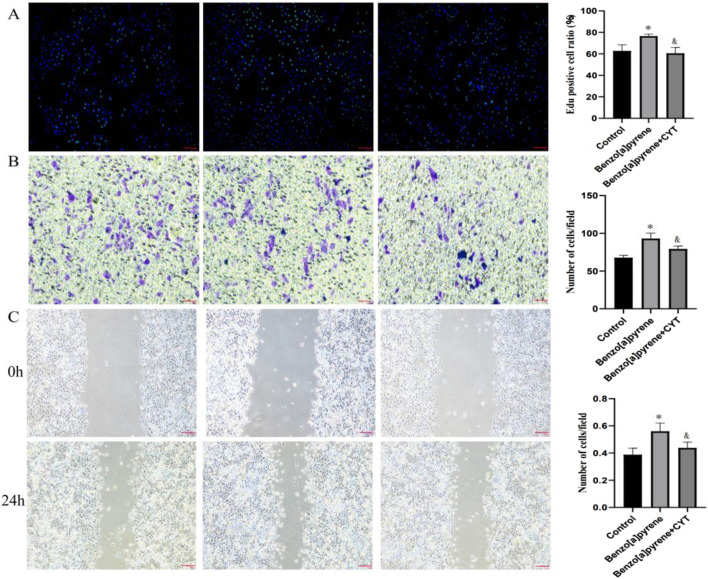
The effects of benzo [a] pyrene and salvianolic acid on prostate cancer cells **(A)**: Edu proliferation experiment; **(B)**: Transwell experiment; **(C)**: Cell scratch test. Note: *: Benzo [a] pyrene vs control, &: Benzo [a] pyrene+CYT vs Benzo [a] pyrene.

Western blot analysis showed that Benzo[a]pyrene treatment significantly increased the protein expression level of PLK1 in PCa cells, while cryptotanshinone treatment effectively reversed this effect (Supplementary Figure S5). Further cell functional experiments demonstrated that ED treatment significantly promoted the proliferation (EdU assay, Figure 5A), invasion (Transwell assay, Figure 5B), and migration (wound healing assay, Figure 5C) of PCa cells; cryptotanshinone intervention similarly reversed these cancer-promoting phenotypes. These results suggest that Benzo[a]pyrene may promote malignant progression of prostate cancer by upregulating PLK1 expression, and cryptotanshinone can antagonize the tumor-promoting effects induced by Benzo[a]pyrene.

## Discussion

EDs are exogenous substances that interfere with the synthesis, secretion, transport, and metabolism of hormones. This disruption can impair physiological balance and development, leading to diverse adverse effects on the reproductive, nervous, and immune systems (Kahn et al., 2020; Yilmaz et al., 2020; Symeonides et al., 2024; Macedo et al., 2023; Singh, 2024). Due to increasing industrialization, EDs are now widespread environmental pollutants. Long-term human exposure through multiple pathways poses a significant health risk, garnering particular concern for its role in the pathogenesis of hormone-dependent cancers such as PCa (Macedo et al., 2023; Singh, 2024; Pang et al., 2024; Feijó et al., 2025). Substantial laboratory evidence confirms a strong link between ED exposure and an increased risk of PCa. For instance, *in vivo* studies demonstrate that prenatal ED exposure in rats can induce transcriptomic and proteomic alterations in the offspring’s prostate, promoting carcinogenesis (Aquino et al., 2023; Aquino et al., 2024; Souza et al., 2025). Complementing these findings, research by Sousa et al. showed that chronic exposure to mixtures of EDs predisposes the rat ventral prostate to neoplasia (Sousa et al., 2023). Similarly, an observational study by Wang et al. (Wang et al., 2024), based on human urinary biomarkers, underscored a positive correlation between ED levels and an elevated risk of PCa.

Although *in vivo* studies have established a strong link between EDs and PCa risk, the field remains constrained by several limitations. The existing literature primarily focuses on the risks posed by individual EDs, offering limited insight into the underlying mechanisms or their role in cancer progression. A comprehensive understanding of the interaction networks and key pathways involved is still lacking (Macedo et al., 2023; Singh, 2024; Pang et al., 2024; Feijó et al., 2025; Aquino et al., 2023; Aquino et al., 2024; Souza et al., 2025; Sousa et al., 2023; Wang et al., 2024). While these findings highlight the need for stricter regulation of EDs, they provide little guidance on effective preventive measures. To address these gaps, we implemented a multi-disciplinary strategy that integrates bioinformatics, machine learning, molecular docking, and *in vitro* assays. This approach allowed us to systematically investigate the ED-PCa relationship, identify key genes and regulatory networks, and screen for natural bioactive compounds that could mitigate the increased PCa risk associated with ED exposure.

In this study, we selected 12 common EDs based on their environmental prevalence and significant potential for carcinogenic human exposure. Subsequent toxicity assessments confirmed their propensity for bioaccumulation, carcinogenic potential, and endocrine-disrupting effects. Our subsequent bioinformatics analysis identified 233 bridging genes that may connect ED exposure to PCa pathogenesis. KEGG and GO enrichment analyses indicated that EDs may promote the development and progression of PCa by modulating processes such as inflammatory response, cell proliferation, hypoxia, hormone secretion, and metabolism. This appears to occur through the disruption of key cancer-related signaling pathways, including Ras, PPAR, and cAMP. These findings underscore the multi-faceted impact of EDs on PCa, acting through multiple targets and pathways. Furthermore, using a robust computational framework of 10 machine learning algorithms across 101 combinations, we identified CD38, MMP11, and PLK1 as reliable prognostic biomarkers, as their expression levels were significantly correlated with patient survival in multiple cohorts.

PLK1, a pivotal serine/threonine kinase, is a master regulator of cell cycle progression, critically involved in mitotic entry, centrosome maturation, and cytokinesis (Parashara et al., 2024; Conti et al., 2024). It is frequently overexpressed in diverse malignancies, including PCa, where its upregulation is strongly associated with tumor progression, metastasis, and therapy resistance—particularly to anti-androgen treatments (Reda et al., 2022; Hoffmann, 2022; Liang et al., 2023; Tang et al., 2020; Zhang et al., 2024; Shin et al., 2019; Patterson et al., 2023). Our findings corroborate this established role, demonstrating that PLK1 is highly expressed in most cancer types and that its overexpression is significantly correlated with poor prognosis in PCa patients. Notably, although the oncogenic function of PLK1 is well-recognized, its connection to EDs has been largely overlooked. Apart from limited studies reporting that BPA and DBP can upregulate PLK1 in other disease models (Zahra et al., 2022; Adir et al., 2017), no research has systematically elucidated its key role in ED-associated carcinogenesis. Our study addresses this gap for the first time: molecular docking results indicated that all 12 tested ED compounds could spontaneously bind to PLK1, with Benzo[a]pyrene exhibiting the strongest binding stability. Furthermore, Western blot analysis confirmed a significant increase in PLK1 protein levels in PCa cells treated with Benzo[a]pyrene. This suggests that chronic, low-dose ED exposure may drive carcinogenic transformation in prostate cells—through sustained PLK1 activation leading to epigenetic remodeling, metabolic reprogramming, and cell cycle checkpoint dysregulation—thereby providing key experimental evidence for the hypothesis that EDs promote PCa development via PLK1 upregulation.

Natural active products, owing to their multi-target and multi-pathway pharmacological properties, have become an important source for anti-tumor drug development (Slika et al., 2022). In recent years, their potential in mitigating health damage caused by environmental pollutant exposure has also gained increasing attention (Watkins et al., 2007; Ghahri et al., 2024). Based on these findings, exploring natural products that can antagonize the carcinogenic effects of EDs is of great significance for developing corresponding protective strategies. In this study, we screened five natural active ingredients targeting the core gene PLK1 using the CTD and validated their binding affinity to PLK1 through molecular docking. Further *in vitro* experimental results demonstrated that cryptotanshinone could effectively inhibit the ED-induced upregulation of PLK1 expression and alleviate its subsequent tumor-promoting effects, providing experimental support for the application of natural products in the prevention and control of ED-related tumors.

Furthermore, it is important to emphasize that while our study identifies PLK1 as a central hub in the EDs-PCa axis, we fully recognize that EDs, as exogenous chemicals, likely exert multi-target biological effects. Our own data support this notion. KEGG enrichment analysis revealed that the shared EDs-PCa targets are significantly enriched in multiple parallel oncogenic and regulatory pathways, including the Ras, PPAR, and cAMP signaling pathways. This implies that alongside the central PLK1 axis, EDs may co-promote tumorigenesis through these parallel networks. This network effect presents two critical possibilities: off-target effects and compensatory mechanisms. On one hand, EDs might independently act on other signaling nodes (e.g., PPARG, PTGS2), and these “off-target” actions could synergistically amplify oncogenic signals with the PLK1 pathway. On the other hand, when the core PLK1 pathway is specifically inhibited (e.g., by cryptotanshinone), cancer cells might activate other compensatory survival pathways (such as the Ras or cAMP pathways indicated by the enrichment analysis) to sustain their malignant phenotype, representing a potential mechanism for drug resistance. This inference aligns with previous studies indicating that EDs like B[a]P can simultaneously disrupt multiple cellular signaling networks (Aquino et al., 2023; Aquino et al., 2024; Souza et al., 2025; Sousa et al., 2023; Wang et al., 2024; Tian et al., 2024; Zhu et al., 2018).

Our results translate a mechanistic discovery into tangible clinical and public health prospects. Firstly, PLK1 emerges not just as a therapeutic target but as a potential biomarker to stratify individuals for their ED-associated PCa risk. This could inform future screening protocols. Secondly, by demonstrating how a natural compound can mitigate this risk, our work provides a direct scientific foundation for nutritional interventions and the development of novel preventatives or adjuvants for at-risk groups or patients. Consequently, this research bridges environmental health science with clinical medicine, offering a roadmap from hazard identification to potential risk reduction.

This study has several limitations that should be acknowledged. Firstly, target identification was primarily based on predictions from bioinformatics databases. Such methods are susceptible to algorithm biases and confidence thresholds, which may introduce inaccuracies or incompleteness in the identified ED targets. Secondly, experimental validation was conducted only for a single endocrine disruptor and a single natural active product. Future work should expand the types and quantities of compounds to enhance the representativeness and generalizability of the results. Furthermore, the current study relies on *in vitro* cell models, which cannot simulate the complete *in vivo* pharmacokinetic processes (such as absorption, distribution, metabolism, and excretion). This limitation is particularly significant given that metabolites of some EDs are also carcinogenic. Finally, although Western blot experiments indicated changes in PLK1 expression levels, the upstream regulatory mechanisms and the specific roles of downstream signaling pathways have not been thoroughly explored and require further molecular experiments for validation.

## Conclusion

Through the integration of network toxicology, multi-omics analyses, a comprehensive assessment of 101 machine-learning algorithms, and subsequent *in vitro* validation, this research definitively establishes PLK1 as a key mediator of EDs-aggravated prostate cancer malignancy. We show that various EDs, including Benzo[a]pyrene, can stably bind to PLK1 and, through the upregulation of PLK1 protein expression, potently drive cancer cell proliferation, migration, and invasion. Importantly, we discovered that natural active products, with cryptotanshinone as a prime example, can effectively antagonize the EDs-induced upregulation of PLK1 and reverse the resulting pro-carcinogenic phenotypes. Our work delivers crucial molecular evidence for understanding EDs-driven cancer mechanisms and proposes targeting the EDs-PLK1 axis as a viable chemo-preventive and therapeutic strategy for prostate cancer.

## Data Availability

The original contributions presented in the study are included in the article/Supplementary Material, further inquiries can be directed to the corresponding author.
