# Supplementary material for: Comprehensive bioinformatics and in vitro studies reveal the carcinogenic role and molecular basis of endocrine disruptors in prostate cancer
Source: Front Cell Dev Biol. 2025 Dec 3;13:1712195. doi: 10.3389/fcell.2025.1712195 (PMC12708592; doi:10.3389/fcell.2025.1712195)
Supplement: Supplementary file 1 [file DataSheet2.docx]

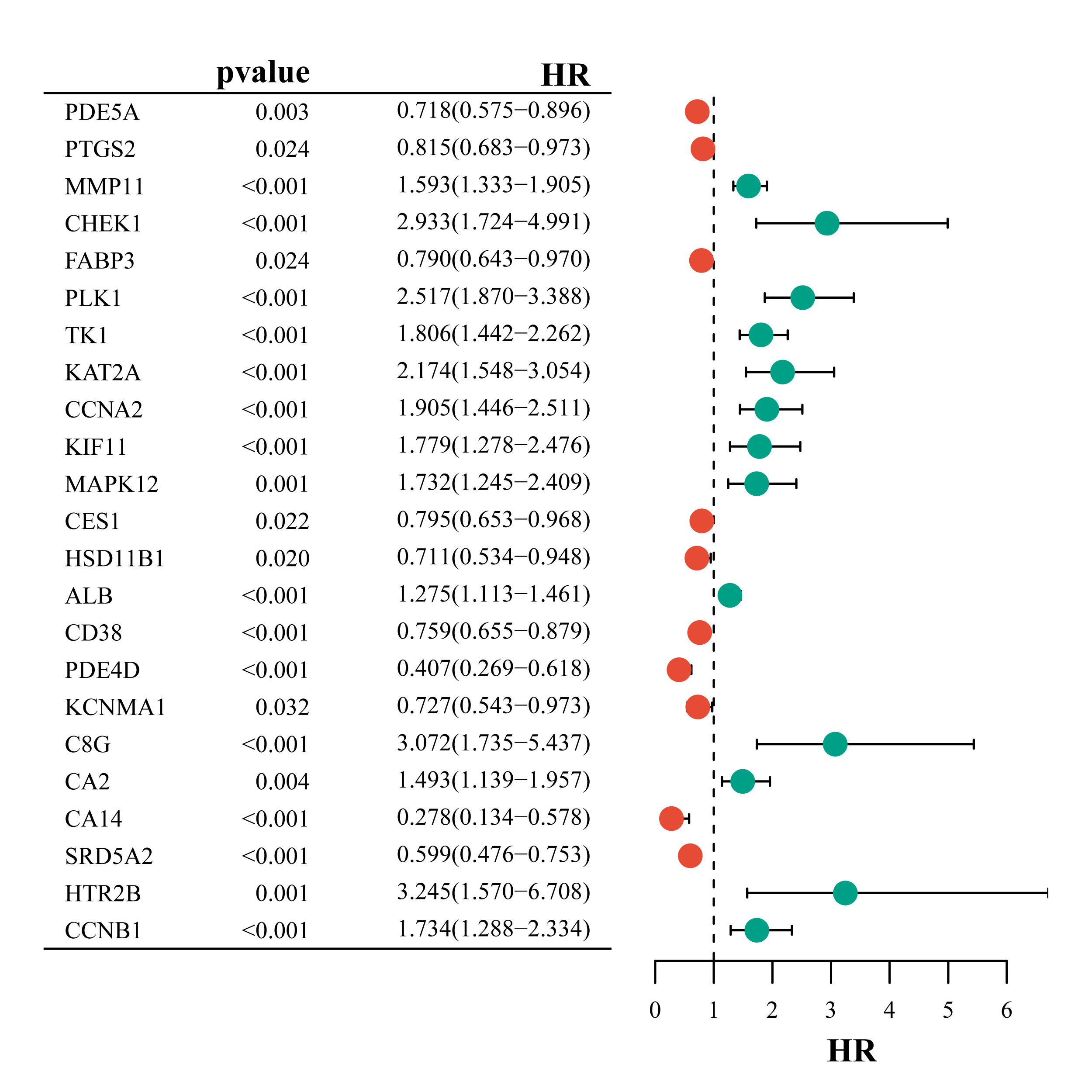


Figure S1: Univariate Cox regression results of 23 common prognostic genes in the TCGA-PRAD and GSE cohorts in the TCGA-PRAD cohort

Note: hazard ratio (HR)


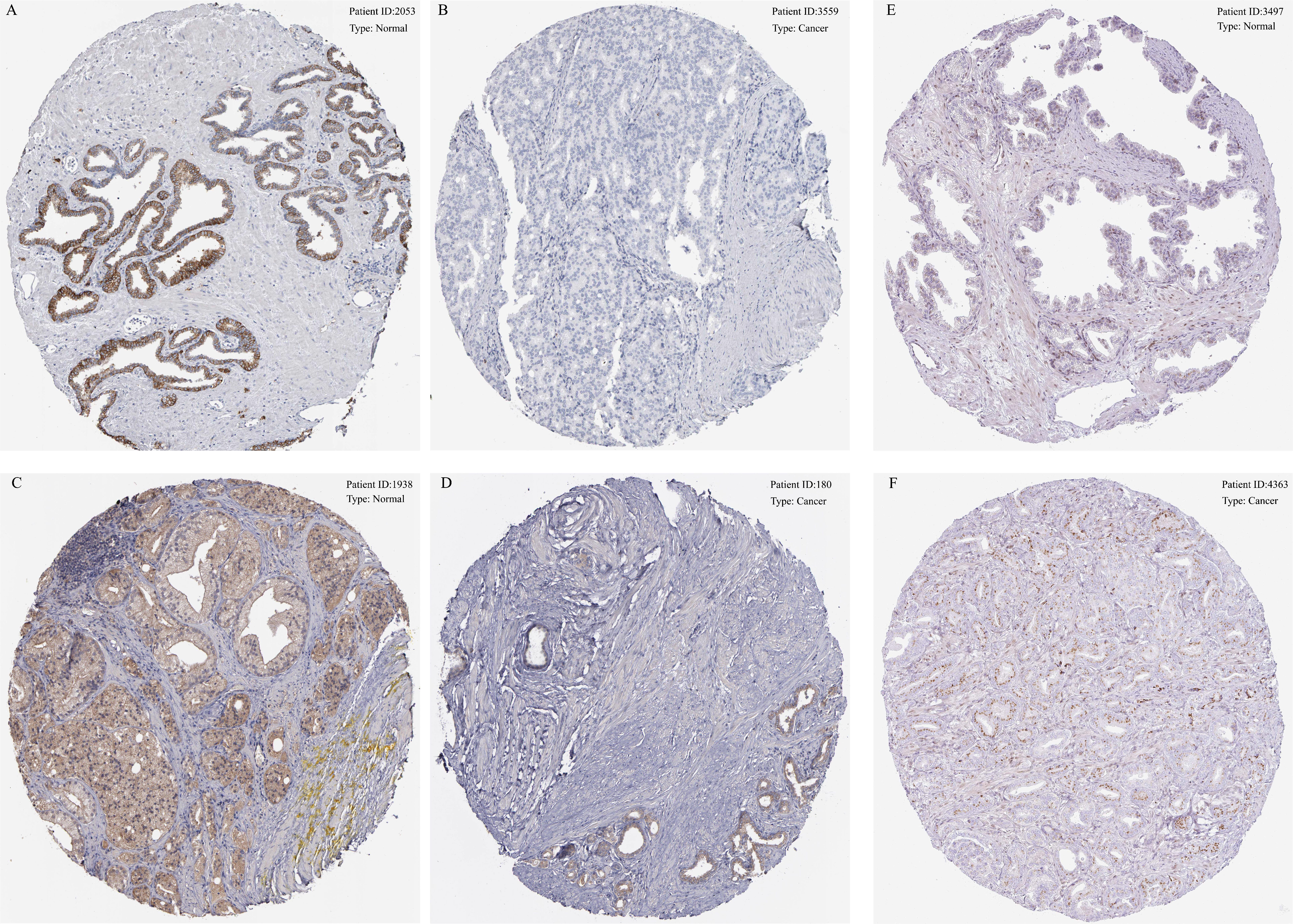


Figure S2: The protein expression of core genes (CD38, MMP11, and PLK1) in normal and tumor tissues.

A/B: CD38; C/D: MMP11; E/F: PLK1; A/C/E: Normal tissue; B/D/F: Tumor tissue


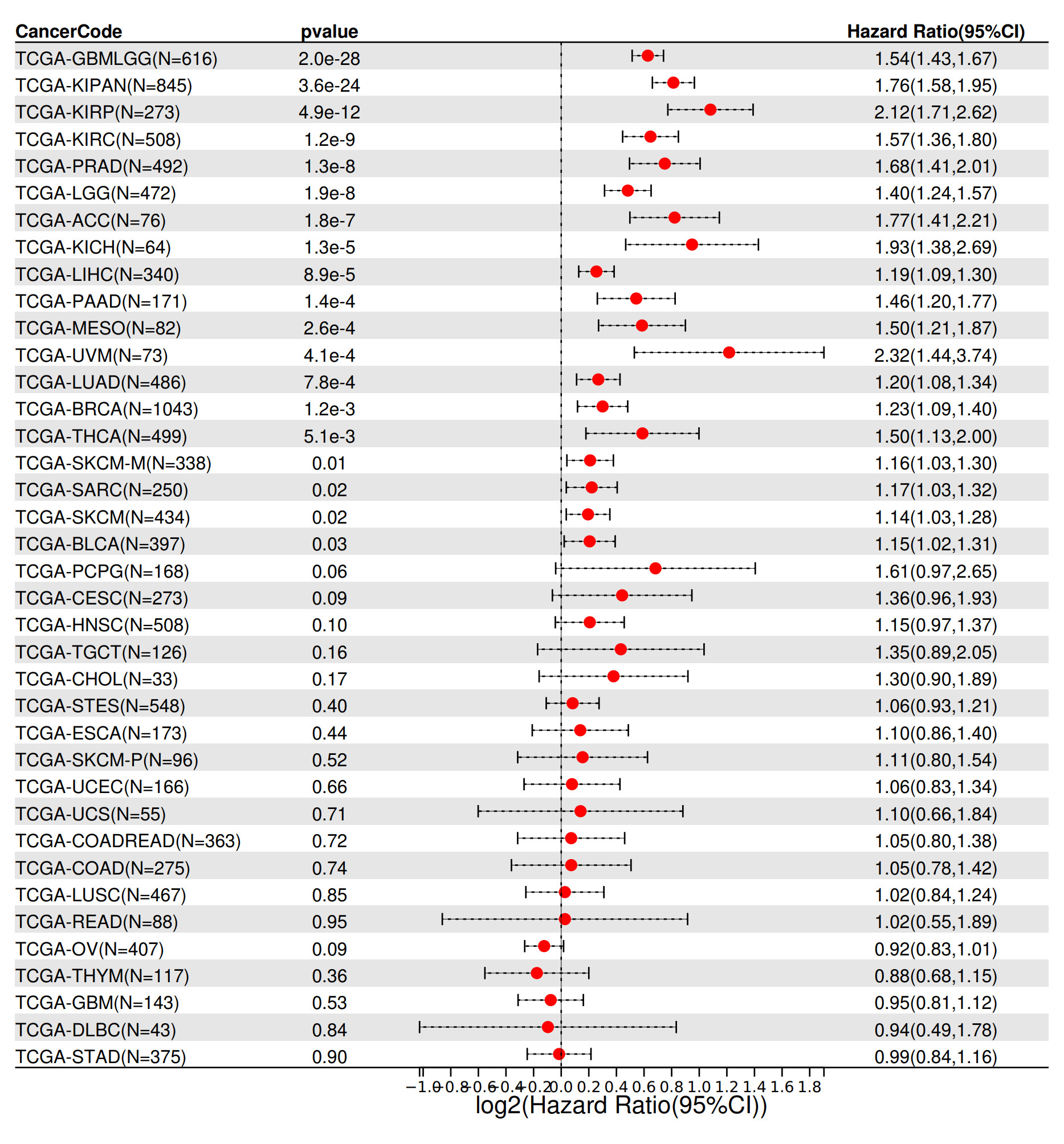


Figure S3: The relationship between poor prognosis of tumors in PLK1 and TCGA data.


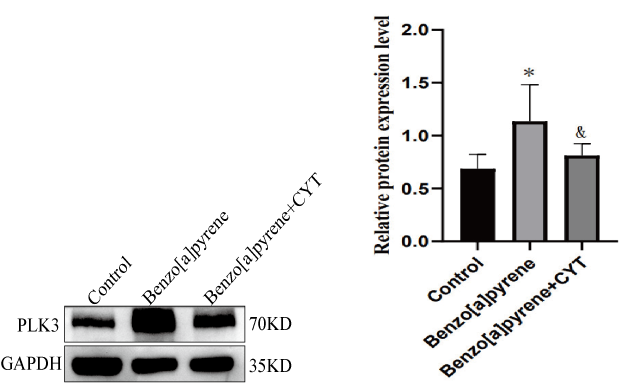


Figure S4: The effects of Benzo [a] pyrene and salvianolic acid on the expression of PLK3 protein.

Statistical significance (p < 0.05) is indicated by symbols: * for Benzo[a]pyrene vs. Control, and & for Benzo[a]pyrene + CYT vs. Benzo[a]pyrene.





Fig S5: Process summary diagram of this research methodology


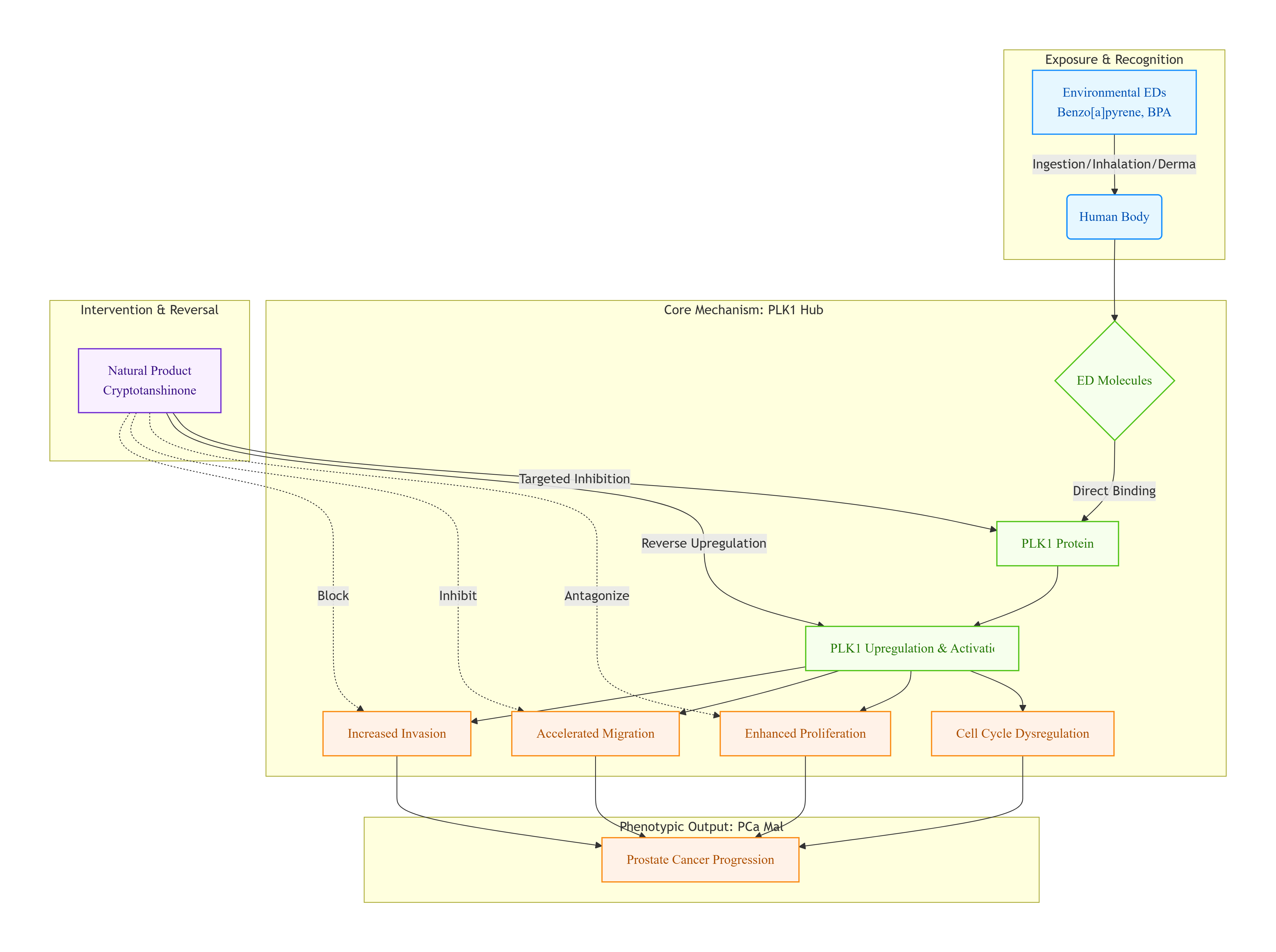


Fig S6: The ED-PLK1-Ca model drawn based on our research results
